# The Clinical Spectrum of Autoimmune-Mediated Neurological Diseases in Paediatric Population

**DOI:** 10.3390/brainsci12050584

**Published:** 2022-04-29

**Authors:** Karol Lubarski, Anna Mania, Sławomir Michalak, Krystyna Osztynowicz, Katarzyna Mazur-Melewska, Magdalena Figlerowicz

**Affiliations:** 1 Department of Infectious Diseases and Child Neurology, Poznan University of Medical Sciences, 27/33 Szpitalna St., 60-572 Poznan, Poland; klubarski@skp.ump.edu.pl (K.L.); amania@ump.edu.pl (A.M.); katarzynamelewska@ump.edu.pl (K.M.-M.); 2 Department of Neurology, Division of Neurochemistry and Neuropathology, Poznan University of Medical Sciences, 49 Przybyszewskiego St., 60-355 Poznan, Poland; swami@ump.edu.pl (S.M.); osztynowiczkr@ump.edu.pl (K.O.)

**Keywords:** child neurology, neuroepidemiology, encephalitis, involuntary movement, neuroimmunology, neuroinflammation

## Abstract

Neurological autoimmune diseases have various origins and pathogeneses. Specific antibodies are associated with paraneoplastic syndromes, other infectious agents, or inherited disorders. We aim to evaluate the relation between the autoantibodies, the chosen symptoms, demographic characteristics, and infection history. We retrospectively analysed 508 children during neurological diagnostics. We investigated serum antineuronal, IgG, IgM anti-ganglioside, and anti-aquaporin-4 in both the serum and cerebrospinal fluid (CSF) anti-cell surface and anti-synaptic protein antibodies in 463, 99, 44, 343, and 119 patients, respectively. The CSF polymerase chain reaction detection of Herpesviridae, enterovirus, B19 parvovirus, adenovirus, and parechovirus involved 261 patients. We included available clinical information and electroencephalographic, radiologic, and microbiological results. The IgM anti-ganglioside antibodies increased the risk of tics and positive symptoms (*p* = 0.0345, *p* = 0.0263, respectively), the anti-glutamic acid decarboxylase particle of paresis (*p* = 0.0074), and anti-neuroendothelium of mutism (*p* = 0.0361). Anti-neuroendothelium, IgM anti-ganglioside, and CSF anti-N-methyl-D-aspartate antibodies were more often associated with consciousness loss (*p* = 0.0496, *p* = 0.0044, *p* = 0.0463, respectively). Anti-myelin antibodies co-occured with Herpes simplex virus (HSV)-2 IgG (*p* = 0.0415), anti-CV2 with HSV-1 IgM (*p* = 0.0394), whereas anti-glial fibrillary acidic protein was linked with past Epstein-Barr virus infection. The anti-ganglioside IgM and anti-myelin particles were bilaterally correlated (*p* = 0.0472). The clinical pictures may overlap, requiring specialistic diagnostics. We noticed the links between the infection aetiology and the specific autoantibody’s positivity.

## 1. Introduction

Autoimmune diseases are intensively studied. The diversified aetiopathological mechanisms remain under continuous research. However, several hypotheses include, e.g., molecular bacterial and viral mimicry, individual susceptibility to autoagression, and other genetic predispositions [1,2].

Specific antibodies appear as a part of paraneoplastic neurological syndromes (PNS) triggered by malignancies [3]. Mentioned rare diseases do not directly occur because of local tumour infiltration or distant metastasising but due to immunisation to antigens common for healthy and cancerous tissues [3,4]. Amongst autoantibodies, the strongest correlation with neoplastic disorders was described for anti-Ma/Ta, antineuronal nuclear antibody (ANNA) type 1 (anti-Hu) and type 2 (anti-Ri), against Purkinje cell cytoplasmic antigen type 1 (anti-PCA1) (anti-Yo) and type 2 (anti-PCA2), amphiphysin, collapsin response mediator protein 5 (CRMP5) (anti-CV2), and anti-Tr antibodies, together named anti-onconeuronal antibodies. Although the clinical course may overleap, specific antibodies also co-occur with certain syndromes [4]. 

Non-paraneoplastic autoimmune neurological syndromes are a large group of diseases in which the immune system abnormally targets the proper central nervous system’s (CNS) antigens, but the underlying trigger remains outside the neoplastic transformation [5]. The particles causing the neurological impairment, aimed against neural antigens, involve anti-myelin, anti-myelin-associated glycoprotein (MAG), anti-neuroendothelium (NET), anti-glutamic acid decarboxylase (GAD), anti-non-myelinated fibres, and anti-glial fibrillary acidic protein (GFAP) antibodies. 

The infective agents, such as Herpesviridae, *Borrelia burgdorferi species* (*B. burgdorferi*), and *Mycoplasma pneumoniae* (*M. pneumoniae*) [2,6], may induce the autoantibodies’ production [6] or facilitate their penetration into the intrathecal compartment [7], leading to clinical manifestations of postinfectious syndromes, probably due to molecular mimicry [1,2].

The study compares current or previous infection indicators, the analysed autoantibodies, and the systemic inflammatory markers in a paediatric population. We planned to observe the autoantibodies’ associations with age and the chosen symptoms incidence in the disease course.

## 2. Materials and Methods

We retrospectively analysed 508 paediatric patients between 2.5 months and 18 years of age suspected of autoimmune disease due to acute or persistent neurologic symptoms. The included participants were hospitalised in a tertiary centre between 3 January 2017 and 2 December 2019. Each child was diagnosed and treated with an individually suited plan.

**Figure 1:**
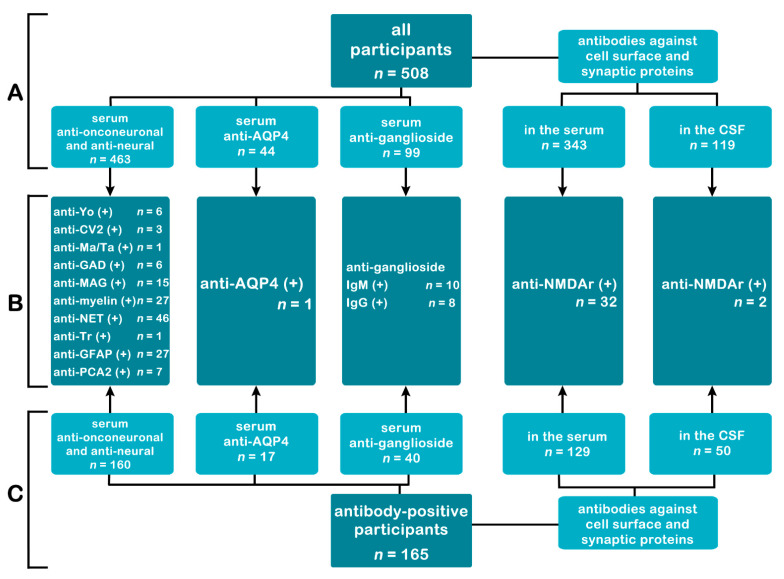
The number of tested patients in subgroups. Part A—the number of patients tested for each group of antibodies in all research participants. Part C—the number of patients tested for each group of antibodies in the antibody-positive subgroup (patients with at least one autoantibody detected). Part B—common to A and C-number of detected antibodies. Abbreviations: anti-Yo—Purkinje cell cytoplasmic antibody type 1; AQP4—aquaporin 4; CSF—cerebrospinal fluid; CV2—collapsin response mediator protein 5; GAD—glutamic acid decarboxylase; GFAP—glial fibrillary acidic protein; Ig—immunoglobulin; MAG—myelin-associated glycoprotein; NET—neuroendothelium; NMDAr—N-Methyl-D-Aspartate receptor; PCA2—Purkinje cell cytoplasmic antigen type 2.

The 463 patients’ sera were tested for nuclear and cytoplasmic antineuronal antibodies (anti-Hu, anti-Ri, anti-Yo, anti-Ma/Ta, anti-PCA2, anti-CV2, anti-Tr, against amphiphysin, GAD, MAG, myelin, NET, GFAP, and non-myelinated fibres), 99 patients for anti-ganglioside antibodies, 343 children were screened for antibodies against cell surface and synaptic proteins (anti-α-amino-3-hydroxy-5-methyl-4-isoxazolepropionic receptor (AMPAr), anti-γ-aminobutyric acid receptor-B (GABA-Br), anti-N-methyl-D-aspartate receptor (NMDAr), anti-1,3-dipropyl-8-phenylxanthine (DPPX), anti-contactin-associated protein-like-2 (CASPR2), anti-leucine-rich glioma-inactivated protein 1 (LGI1)), and 44 patients were tested for anti-aquaporin-4 (AQP4) antibodies. We screened 119 children’s cerebrospinal fluid (CSF) for antibodies against cell surface and synaptic proteins. All mentioned analyses used standardised commercial kits (EUROIMMUN, Luebeck, Germany). All children in whom the paraneoplastic antibody was detected underwent subsequent screening for underlying tumours. Figure 1 depicts the number of tested patients.

The CSF collected by lumbar puncture underwent a routine test with a standard laboratory analyser. The reference values (RV) were as follows: For CSF leukocyte count (CSF-L) RV ranged 0–5 cells/uL, and for CSF protein (CSF-P) concentration stayed within 15–45 mg/dL. The IgG index upper level of normal (ULN) equalled 0.75. The 261 patients underwent polymerase chain reaction panel detecting the genetic material of herpes simplex virus (HSV)-1, HSV-2, varicella-zoster virus (VZV), Epstein–Barr virus (EBV), cytomegalovirus (CMV), human herpesvirus (HHV)-6, HHV-7, B19 parvovirus, enterovirus (EV), adenovirus (ADV), and parechovirus (PV) (Fast Track Diagnostics, Junglinster, Luxembourg) in CSF. We included available antimicrobial results involving antibodies against HSV-1, HSV-2, EBV, CMV, EV, *B. burgdorferi*, *M. pneumoniae*, and tick-borne encephalitis virus (TBEV). The antistreptolysin O (ASO) ULN equalled 150 IU/mL. We analysed electroencephalography and radiological findings in magnetic resonance imaging (MRI) and computed tomography (CT). The participants’ final diagnoses stand in Table 1.

We considered the frequency of consciousness loss, involuntary movements, paresis, focal symptoms, speech or sight aberrances, headaches, meningeal signs, and urination or defecation disorders. Moreover, we analysed symptoms’ association with the particular autoantibody.

We provided the statistical assessment with Statistica 13.3 (TIBCO Software Inc., 2017, Palo Alto, CA, USA). Depending on the data distribution and the number of groups, we used the χ^2^, the Mann–Whitney U (MW) test. A *p*-value of less than 0.05 we considered statistically significant.

The Institutional Ethical Committee stated that formal approval and informed consent is not required due to the retrospective character of the study, including anonymised data analysis from clinically justified procedures.

## 3. Results

Our study group included 508 participants with a median age of 8.9 years old. We compared the parameters separately in the whole population (p1) and among autoantibody-positive patients (p2). We found that patients with anti-CV2 (2.5 vs. 9.0 [y/a], p1 = 0.0328) and anti-PCA2 (4.7 vs. 9.1 [y/a], p1 = 0.0095) have significantly lower age than those without either particle; however, the anti-Yo-positive patients were older than those without mentioned particle (13.3 vs. 8.9 [y/a], p1 = 0.0208). The same conclusions appeared in analyses within the autoantibody-positive population (2.5 vs. 8.9 [y/a], p2 = 0.031; 4.7 vs. 9.0 [y/a], p2 = 0.0083; 13.3 vs. 8.7 [y/a], p2 = 0.0203, respectively). We noticed that anti-myelin-positive children prevail between 3 and 6 y/o (p1 = 0.0152; p2 = 0.0152).

In routine CSF examination, a significantly higher protein concentration occurred in a group with CSF anti-NMDAr antibodies than in children without them (p1 = 0.0499). What is more, both in whole and the autoantibody-positive populations, patients with anti-myelin antibodies had significantly lower glucose concentrations than children without the particles (p1 = 0.0054; p2 = 0.0094, respectively). The median and IQR of analysed values appear in Table 2.

In the antineuronal autoantibody-positive group, patients suffered most frequently from consciousness alternation (25.6%), involuntary movements (24%), and headaches (22.5%). A large share presented focal signs (20.2%) and sight deterioration (17.1%). The symptoms’ frequency did not statistically differ from autoantibody-negative patients. Separately, patients with serum anti-NET, anti-ganglioside IgM, and CSF anti-NMDAr antibodies had a significantly higher consciousness loss incidence than children without mentioned immunoglobulins, both in the total research population (p1 = 0.0496, p1 = 0.0044, p1 = 0.0463, respectively) and in the autoantibody-positive patients’ group (p2 = 0.0185, p2 = 0.0258, p2 = 0.0367, respectively). Moreover, children with anti-MAG antibodies had a lower cognisance alternation rate (p1 = 0.0159; p2 = 0.0229). In addition, anti-ganglioside IgM positive patients statistically more often experienced involuntary movements (p1 = 0.0345), obsessive-compulsive disorder (OCD) (p1 = 0.0263), or positive symptoms (p1 = 0.0263). Similar observations did not apply to other autoantibodies. Our report suggests that the children with anti-GAD are more often diagnosed with varying severity paresis (p1 = 0.0074; p2 = 0.0078). Another result indicated that defecation problems might be significantly more frequent in the anti-GAD positive population (p2 = 0.0375); however, in our study, only one out of six anti-GAD positive children suffered from the mentioned ailment. We noticed mutism in 6.5% of patients with anti-NET antibodies. Compared to patients without such antibodies, it appeared to be a significant symptom (p1 = 0.0361). The CSF anti-NMDAr immunoglobulins’ presence was associated with headache in our population (p1 = 0.0271; p2 = 0.0294). The frequency of focal and meningeal signs, balance, gait, sight disturbances, seizures, and impaired urination between patients with and without specific autoantibody did not differ significantly. We placed the complete data in Table 3.

The population with at least one autoantibody showed a higher anti-M. pneumoniae IgM positivity (p2 = 0.01; OR = 3.7623 CI95% (1.3575–10.4274))—especially anti-myelin antibodies (p2 < 0.01; OR = 5.3333 CI95% (1.5701–18.1165)), which is associated with HSV-2 IgG presence (p2 = 0.04; OR = 9.0952, CI95% (1.5505–53.3528)). The anti-CMV IgM appeared in all CSF anti-NMDAr positive patients. Further analyses confirmed the significance in the whole research group and the limited autoantibody-positive cohort (p1 = 0.01; p2 = 0.01). Previous EBV infection appears as an important anti-GFAP antibody production factor. In our cohort, anti-EBV viral-capsid antigen (VCA) IgG and anti-Epstein–Barr nuclear antigen (EBNA) antibodies’ prevalence resulted in the significant connection with anti-GFAP positivity (p1 < 0.01; OR = 6.6909, CI95% (1.5321–29.2197) and p1 = 0.02; OR = 3.4563 CI95% (1.1361–10.5155)), respectively). We placed the complete data in Table 4.

The paired autoantibodies’ analysis revealed the link between the anti-ganglioside IgM and anti-NET immunoglobulin coexistence (p1 = 0.047; OR = 6.0857 CI95% (1.1943–31.0118)). Other coappearing autoantibody pairs did not show statistical significance in our research.

The PCR CSF detection of neurotrophic viruses found specific HHV-7, EBV, EV, ADV, B19 parvovirus, HSV-1, HSV-2, and HHV-6 nucleic acids in 5 (1.9%), 4 (1.5%), 2 (0.8%), 2 (0.8%), 2 (0.8%), 1 (0.4%), 1 (0.4%), and 1 (0.4%) patient, respectively. Further statistical analyses with χ^2^ did not bring correlation with any autoantibody.

## 4. Discussion

### 4.1. Anti-NET Antibodies

Anti-NET antibodies appear in rheumatic diseases [8], demyelinating autoimmune disorders including multiple sclerosis (MS), peripheral polyneuropathies [9], and paraneoplastic syndromes [10]. The targets spread in cerebral and nerve-associated vessels of the peripheral (PNS) and CNS. Chronic diseases may induce autoantibodies, which later, due to injury to the blood–brain barrier (BBB), may penetrate the intrathecal compartment [9]. The neuroendothelial cells participate in haemostasis and inflammatory response [11,12]; in addition, the cognitive impairment correlates with antibodies titre [13]. Our results partially fit the data in other research, in which seizures were observed in 33% of patients, upper or lower motor neuron involvement in 27% and 22% of patients, respectively, cranial nerve impairment bothered half of all patients, and cerebellar syndrome occurred in 65% of them [14]. The positive psychiatric symptoms’ incidence was comparable to other autoantibodies and autoantibody-negative patients. In contrast, OCD exceeded the share amongst autoantibody-negative (6.5% vs. 3.8%) or participants with any autoantibodies detected (6.5% vs. 4.2%). However, the statistical significance was not found. In contrast to previous studies, we did not observe an increased cardiovascular incident rate in autoantibody-positive patients—ischaemic stroke appeared in one patient [13].

The pairwise tests revealed significant anti-NET and anti-ganglioside IgM coexistence. The publications describe the in vitro direct anti-monosialotetrahexosylganglioside antibodies’ cross-reaction on microvascular endothelial cells in neuropathy, leading to disruption of the barrier between vessels and nerves, causing the exposure of other antigens [15]. However, the hypothesis concerns the peripheral nervous system and does not reflect the clinical reports.

### 4.2. Anti-GFAP Antibodies

The antibodies to GFAP cause inflammations involving the brain, cerebellum, meninges and brain stem, resulting in encephalopathy [16]. The clinical picture contains severe headaches, consciousness deterioration, and involuntary movements and less frequently partial paresis, ataxia, and other walking difficulties. Patients show memory loss, psychotic episodes, or autonomic involvement such as urine and stool incontinence or retention [17]. Fang et al. estimate that every fifth anti-GFAP-positive patient requires prolonged immunosuppression to sustain the remission. In our population, the most significant differences concern consciousness deterioration (18.5% vs. 68.8%), headaches (22.2% vs. 81.3%), and involuntary movements (18.5% vs. 33.3%), whereas paresis frequency was similar in both reports (18.5% vs. 18.8%) [18]. The cited authors selectively analysed an adult population with autoimmune disorders, whereas we based our research on a heterogeneous paediatric cohort, including children with infective or unclear aetiology. In the case series, the authors described two children who presented gait impairment and nystagmus. What is more, the first diagnosed with meningoencephalitis showed dysautonomia, bladder dysfunction, and cerebellar syndrome, whereas the second suffered from headaches and consciousness alternation, followed by extrapyramidal syndrome [19]. Our four anti-GFAP-positive participants had other autoantibodies detectable. We did not observe children with positive psychiatric symptoms in our population, and only one patient experienced autonomic urination impairment. Our data revealed a significantly higher anti-GFAP incidence in children with detected anti-VCA EBV IgG or anti-EBNA IgG. However, the literature lacks data on the correlation between anti-GFAP and infectious agents.

### 4.3. Anti-Myelin Antibodies

Autoantibodies against myelin compounds participate in various clinical syndromes such as cerebellar dysfunction, extrapyramidal symptoms, motor neurons, and vision impairment. The involvement of cranial nerves differs between reports [14,20]. The anti-myelin particles appear in physiological protective reactions to CNS trauma but also occur in patients with a past oncological history or accompany primary autoimmune disorders and rheumatic diseases [14,21,22]. Our anti-myelin-positive patients suffered most frequently from involuntary movements, paresis, or sight deterioration and consciousness alternation.

Myelin oligodendrocyte glycoprotein (MOG) occurs exclusively in the CNS [23]. The antibodies against MOG predominantly affect an optic nerve and a spinal cord, accompanying difficulties with speech, swallowing, vision, and oculomotor function. The research in the paediatric population correlated the anti-MOG antibodies with neuromyelitis optica (NMO) and transverse myelitis (TM) in monofocal and polyfocal forms. The antibody frequently appears in acquired demyelinating diseases in childhood—especially in the younger population presenting acute disseminated encephalomyelitis (ADEM). The authors also concluded that anti-MOG-positivity during the acute phase in almost 50% is transient, as well as with non-MS disease course [24]. The positivity concerns 20% of patients with NMO and TM but twice more with ADEM [25,26]. Moreover, anti-MOG persistent positivity brings relapse risk 3–5 times greater [27]. The HSV may cause peripheral neuropathies, including facial nerve palsy [28]. We found a correlation between anti-myelin positivity and anti-HSV-2 IgG particles, but previous studies suggest anti-MOG antibodies’ peripheral presence preceding such infection. The viral disease impairs the BBB, enabling the circulating autoantibodies to conjugate with CNS and trigger symptoms [29]. Literature also confirms that influenza and EBV participate in the pathogenesis of anti-MOG ADEM [30], having a predictive value in the relapsing multiphasic course risk [27]. The viral infections preceding the NMO confirm that infectious diseases facilitate autoantibodies penetration to the intrathecal compartment [6]. The animal model independently proved the HSV-1 potential to trigger encephalitis and multifocal demyelinated CNS lesions [6]. Zheng et al. noticed the cross-reactivity between MOG and CMV in the rat experimental autoimmune encephalomyelitis (AIE) model [31]. However, postinfectious anti-MOG positive NMO appears after bacterial infections (e.g., *B. burgdorferi* and *Mycobacterium tuberculosis* (*M. tuberculosis*)) [7,32]. We found the association with M. pneumoniae IgM that fits previous reports hypothesising the molecular mimicry triggering anti-myelin antibodies production and immunocomplexes deposition causing perivenular inflammation [1,26]. Christie et al. confirmed the cross-reaction with galactocerebroside C in 38% of *M. pneumoniae* encephalitic patients [33].

### 4.4. Anti-MAG Antibodies

The anti-MAG antibodies are responsible for various neuropathies. Several kinds of research successfully confirmed the pathological role of the particles [34], e.g., the passive transfer of human-derived anti-MAG antibodies to chickens. The investigation revealed the development of a similar pathophysiological pattern of deposits in myelin sheets and structural change of the layers [35]. The most typical course begins with numbness and upcoming paraesthesia, leading to distal, symmetrical sensory impairment, mainly due to the injury of myelinated fibres. Motor involvement with muscle weakness and ataxia rarely happen [36]. In our population, patients most frequently experienced involuntary movements, sight deterioration, and gait impairment in 40%, 26.7%, and 20% of cases, respectively. Compared to the available report in which balance impairment involved 17.7% of adult patients, the incidence reached 13.3% in our population. We did not observe cranial nerve involvement or epileptic seizures, which bothered 50% and 66% of cited research patients. The same authors detected motor neurons involvement in over 34% and myopathy in 60% of participants [14]. Findings associated with anti-MAG antibodies more typical for the paediatric population include ataxia after chicken pox [37] and their presence in 70% of patients diagnosed with autism [38].

The detected link between anti-MAG positivity and antecedent CMV infection was described in a previous study in which a CMV DNA occurred in 88% of anti-MAG positive patients with chronic neuropathy. The mechanism remains unknown, but molecular mimicry was suspected [39]. However, several works undermine the correlation and suggest the latent CMV reactivation, but the acquirable data cannot justify anti-MAG production [40]. In addition, Lunn at. al. failed to confirm the hypothesis in patients with peripheral neuropathy and paraproteinemia [41].

### 4.5. Anti-PCA2 Antibodies

The anti-PCA2 is a partially characterised onconeuronal antibody associated with small-cell lung carcinoma (SCLC). In our research, the tics dominated amongst children’s symptoms; only one suffered from the walking impairment or experienced a consciousness decrease. However, the previous publications describe a wide range of symptoms potentially associated with the mentioned antibody due to encephalitis, myelitis, or neuropathy. Cognitive impairment appears as a short-term memory decrease, disorientation, and consciousness clouding. The paraneoplastic syndrome may manifest with cerebellar ataxia, regional paraesthesia, spastic paresis, or myasthenic disorder [42,43].

### 4.6. Anti-GAD Antibodies

The anti-GAD antibodies participate in autoimmune DM pathogenesis [44]. The neurological syndromes are rare, have marked female predominance, and appear in patients with anti-GAD detectable in CSF [45]. They involve cerebellar ataxia, stiff-person syndrome, AIE, and epileptic episodes resulting from impaired GABAergic transmission [46]. Paediatric data suggest that high anti-GAD titres provoke temporal-based focal seizures, psychiatric symptoms, and cognitive decrease, manifesting in remembrance difficulties and developmental slowdown [5]. On the other hand, up to 1.7% of healthy and 5% of neurological patients with different aetiology are anti-GAD positive [47]. We observed pareses and impaired defecation in our population in 66.7% and 16.7% of participants, respectively. The 16.7% of patients also experienced balance difficulties. Honnorat et al. described gait impairment in all patients; however, the study focused on patients with cerebellar involvement. The seizure and visual symptoms rates were lower, reaching 33.3% vs. 53% and 16.7% vs. up to 86%. We noticed a similar consciousness alternation frequency than previously described for anti-GAD-associated cerebellitis. We did not note memory deterioration, which appeared in 67% of patients in the cited research [44]. In research concerning children with AIE, authors described a change of behaviour and alternated consciousness in all three anti-GAD positive patients; two presented hallucinations and sleepiness, and one patient had an autonomic disorder or tremor [48].

### 4.7. Anti-Yo Antibodies

Anti-Yo belongs to well-described autoantibodies associated with ovarian and breast cancer, but 2–8.5% of non-oncologic patients occur positive [4,49]. The most typical clinical manifestation bothering 90% of cases includes cerebellar degeneration; however, brainstem encephalitis or peripheral neuropathy happens rarely [49]. In our non-oncologic population, we diagnosed paresis in two patients; in addition, two experienced consciousness deterioration and one patient had tics. In literature, varied ataxia and nystagmus concerned all patients; peripheral neuropathy appeared in 47% and cognitive impairment in 18% of paraneoplastic syndrome cases [50]. In the paediatric population, anti-Yo-related paraneoplastic syndrome with typical PCD, including ataxia, speech impairment, and vertigo, coappear with Hodgkin’s lymphoma; in children without malignancy, the anti-Yo positivity rate increases in patients diagnosed with attention deficit hyperactivity disorder [51].

### 4.8. Anti-CV2 Antibodies

The anti-CV2 antibody accompanies SCLC and, less often, thymoma, but 4–17.5% of non-oncologic patients show positivity [4,49]. Clinical manifestations include limbic encephalitis, cerebellar degeneration with ataxia and involuntary movements, encephalomyelitis, sensory and sensorimotor neuropathy, gastrointestinal pseudo-obstruction, and ocular inflammation [4,52]. Among our patients with anti-CV2 in serum, the one experienced headache and gait impairment; the other, with multi-antibody positivity, showed mutism, whereas the third with autism was asymptomatic. Psimaras et al. described peripheral neuropathy in 39%, cerebellitis in 13.4%, and limbic encephalitis in 4.3% of patients with anti-CV2 in CSF [49]. 

Interestingly, significant anti-CV2 positivity appeared amongst children with anti-HSV-1 IgM. A similar association was not previously described, nor with another infectious agent. The analysis requires further research because of the low positive patient quantity and potential statistical bias.

### 4.9. Anti-Tr Antibodies

Antibodies against Tr protein are partially characterised paraneoplastic molecules predominantly observed in Hodgkin lymphoma, but 11% of positive patients had no detectable neoplasm [4]. Amongst analysed children, we detected the anti-Tr antibodies in a single patient with autism and epilepsy hospitalised due to transient consciousness impairment. What is more, the boy also produced anti-GFAP antibodies. Typically, anti-Tr antibodies cause PCD or limbic encephalitis [49], but in children, paraneoplastic syndromes are casuistic, including vision impairment, ataxia, tremor, and muscular hypotonia [53]. Characteristic ataxia may appear as an isolated syndrome or combine with encephalopathy and peripheral neuropathy [42].

### 4.10. Anti-Ma/Ta Antibodies

This well-described onconeural antibody appears in germinal testicular tumours or non-SCLC and less frequently in other solid neoplasms. The main clinical presentations include limbic or brainstem encephalitis and PCD, with their frequencies assessed for 58%, 21%, and 16%, respectively [49]. Patients with underlying tumours constitute 58–96% of positive participants [4]. They experience consciousness alternation, cataplexy, sleep disorders and diencephalic disorders [54]; children are more prone to focal seizures, behaviour change, or speech and muscle tone impairment [5]. In paediatric non-paraneoplastic AIE typical symptoms include behavioural change and, speech and consciousness impairments, but sometimes positive psychiatric symptoms appear [48]. In our research, the boy with anti-Ma/Ta antibody suffered from blurred vision and headache associated with optic nerve inflammation, foretelling MS.

### 4.11. Anti-NMDAr Antibodies

Autoantibodies against NMDAr most frequently cause AIE. In the general population, 58% of patients are diagnosed with ovarian teratoma [55], but antecedent infection appears typically amongst children. The best-described infective anti-NMDAr encephalitis triggering factor is HSV-1; the other viruses’ roles are ambiguous [2,6,7,56]. Our research suggests the link between CSF anti-NMDAr and CMV IgM positivity. The clinical spectrum includes dyskinesis, choreoathetosis, dystonic posturing, and rigidity. Psychiatric symptoms such as insomnia, paranoia, anxiety disorders, and cognitive deterioration appear less frequently [3,42]. Our patients with CSF anti-NMDAr antibodies presented consciousness loss and severe headaches. Imbalance and motion problems probably resulted from generalised weakness. Amongst patients with those antibodies in serum, 13% experienced deterioration in cognitive functions. However, the most common complaint was vision impairment (50%), and 21% suffered from involuntary movements; psychiatric disorders appeared in 13% and paresis in 16% of cases. The results partially cover the previously observed clinical pictures of paediatric AIE; however, decreased consciousness occurred in less than 50% of cases. The report describes behavioural alternation in all patients, speech disorder in 73%, facial dyskinesias in 64%, and hallucinations in 18% of children [48].

### 4.12. Antiganglioside Antibodies

Gangliosides, crucial for signal transition, spread on neurons’ surfaces. The antibodies against them participate in chronic and acute neuropathies’ pathogeneses; the role in paediatric idiopathic epilepsy needs further research [57]. Antiganglioside antibodies may act as primary or secondary aetiological factors. Although specific targets characterise particular neuropathies, we analysed them collectively. 

IgM autoantibodies appear in multifocal motor neuropathy, ataxic neuropathy, and ophthalmoplegia [36]. In our population, 50% experienced involuntary movements, 20% positive symptoms and OCD, whereas 40% experienced at least transient consciousness loss. We proved those symptoms’ statistical predominance in comparison to autoantibody-negative patients. Moreover, 20% experienced paresis, and 10% experienced gait impairment. 

The children who presented IgG antibodies suffered more frequently from muscle weakness and gait impairment. Nevertheless, less often exhibited symptoms included consciousness alternation or positive psychiatric symptoms. In the literature, IgG anti-ganglioside antibodies link with specific symptomatic disorders or diseases with other known aetiology, e.g., Guillain–Barre syndrome (GBS), MS, and Alzheimer’s or Parkinson’s disease [57]. Research has widely described the association of GBS with previous CMV, EBV, and *M. pneumoniae* infections [58], but our analyses did not bring sufficient evidence to confirm the findings in our population.

### 4.13. Anti-Aquaporin-4 Antibodies

In our research, we detected one patient diagnosed with non-specific stress-associated vision impairment. She reported headache, sight deterioration, presented involuntary movements, and balance impairment. The NMO spectrum disorder most frequently appears as myelitis (84%) and optic neuritis (63%). A brain, brainstem, and area postrema are involved in around 15% of cases. The disease may occur idiopathically or after viral infection with a tendency to relapse [59,60]. Anti-AQP4 antibodies typically manifest with transient vision loss, peripheral sensory and motor impairment, and paroxysmal movements episodes in 20% of patients [42].

## 5. Conclusions

The paper presented the diseases’ manifestations and sought pathogenesis. The clinical pictures partially differed from courses described in adult patients. The main reasons may involve involve a lower incidence of malignancies and a higher prevalence of infectious diseases in children. The protocol involving autoantibodies’ assessment in non-specific neurologic diagnostics might contribute to the detection of pathogenic particles in the pre-clinical phase, enabling the close patient’s observation and prompt treatment in the early stage.

The main drawback resulting from a retrospective study is the difficulty in comparing symptoms between patients and low quantity in subgroups decreasing the tests’ strength. The other source of bias remains the inclusion of patients in different stages of diseases. This aspect concerns both infective and non-infective aetiologies; however, in the first case, the serological testing would be helpful, especially after the acute phase. The report’s objectivity could be improved by the standardised protocol used for patient anamneses and examinations.

## Figures and Tables

**Table 1:** Clinical description of patients divided due to autoantibody presence.

Detected Antibody	Clinical Presentation
anti-CV2, anti-myelin, anti-NET, serum anti-NMDAr (*n* = 1)	− AIE, post-inflammatory generalised epilepsy
anti-MAG, anti-NET, anti-ganglioside IgM (*n* = 1)	− Kawasaki disease
anti-NET, serum anti-NMDAr (*n* = 4)	− AIE, vision disturbance, generalised weakness − AIE, optic chiasm inflammation, sudden visual acuity deterioration, motor coordination decrease − Motor tics − Generalised epilepsy, ASD
anti- NET, anti-GFAP (*n* = 3)	− Autoagression, developmental delay, paroxysmal screaming, behaviour disturbance − Vision disturbance, longsightedness, short-term consciousness deteriorations in history − OCD, streptococcal allergisation; PANDAS susp.
anti-myelin serum anti-NMDAr (*n* = 2)	− Transient motor and sensory aphasia, vision disturbance; autoimmune hypothyroidism − Tics, hyperintense MRI lesion; CANS susp.
anti-ganglioside IgM, anti-ganglioside IgG (*n* = 2)	− Unilateral ptosis, left-sided strabismus − Encephalitis, positive symptoms, hallucinations, nonsensical speech
anti- NET, anti-ganglioside IgM (*n* = 2)	− Autoimmune polyneuropathy, selective mutism, limb pains, gait abnormality − Rolandic epilepsy
serum anti-NMDAr, CSF anti-NMDAr (*n* = 2)	− ADEM, fever, headache, consciousness deterioration, confusion − Encephalomeningitis, status epilepticus headache, weakness
anti-ganglioside IgG, serum anti-NMDAr (*n* = 1)	− Spastic quadriparesis, claudication, lower limbs pain, sensory disturbance
anti-GFAP, anti-Tr (*n* = 1)	− Autism, epilepsy; unspecified autoimmune CNS disorder
anti- NET, anti-myelin (*n* = 1)	− Chronic vocal and motor tic disorder; Tourette’s syndrome
anti-GFAP, anti-GAD (*n* = 1)	− Cerebellitis, cerebellar ataxia, unspecified connective tissue autoimmune disease
anti-GFAP, serum anti-NMDAr (*n* = 1)	− Motor tics, vision disturbance, streptococcal allergisation; infection-associated deteriorations
anti-MAG, anti-PCA2 (*n* = 1)	− Cerebral palsy, symptomatic generalised epilepsy
anti-ganglioside IgM, serum anti-NMDAr (*n* = 1)	− Chronic motor tics with IVIG-dependent improvement
anti-NET (*n* = 34)	− Epilepsy (*n* = 7) − PDD (*n* = 5) − Neuropathy - GBS (*n* = 1) - axonal (*n* = 1) - CMT polyneuropathy (*n* = 1) - IX, X, XII nerves neuropathy (*n* = 1) − Encephalitis: - enteroviral (*n* = 1) - autoimmune (*n* = 1) - unspecified (*n* = 1) - encephalocerebellitis (*n* = 1) − Tics (*n* = 2) − Behaviour disorder (*n*= 2) − Autoimmune diseases: - SM (*n* = 1) - JIA (*n* = 1) − PANDAS (*n* = 2) − Muscular hypotonia (*n* = 1) − Acquired toxoplasmosis, migraine (*n* = 1) − Ischemic stroke, developmental delay (*n* = 1) − Bilateral optic nerve atrophy, epilepsy with tonic-clonic seizures (*n* = 1) − Central vertigo with IVIG-dependent improvement (*n* = 1) − Anisocoria, hypodensive CNS CT lesions, meningeal hernia (*n* = 1)
anti-myelin (*n* = 23)	− PITAND: - associated with *M. pneumoniae* (*n* = 2) - PANDAS (*n* = 2) - PANDAS susp. (*n* = 2) - undefined (*n* = 1) − Encephalitis: - encephalomyelitis (*n* = 1) - pneumococcal encephalomeningitis (*n* = 1) - VZV encephalitis (*n* = 1) - undefined (*n* = 1) − Epilepsy (*n* = 3) − Neuropathy: - polyneuropathy (*n* = 2) - recurrent VII nerve neuropathy (*n* = 1) − Tics (*n* = 2) − Headache (*n* = 2) − CANS, PDD, tics; epileptic seizures in history (*n* = 1) Myopathy, streptococcal allergisation (*n* = 1)
anti-GFAP (*n* = 21)	− Epilepsy (*n* = 6) − Encephalitis - ADEM (*n* = 1) - autoimmune (*n* = 1) - autoimmune cerebellitis (*n* = 1) - enteroviral encephalomeningitis (*n* = 1) − PDD (*n* = 3) − Neuropathy: - autoimmune demyelinating process (*n* = 1) - VII nerve neuropathy (*n* = 1) − PANDAS (*n* = 2) − Headache (*n* = 1) − Gait abnormalities in conversion disorder (*n* = 1) − SMA type 1 (*n* = 1) − Influenza A H1N1, acute otitis externa (*n* = 1)
anti-MAG (*n* = 13)	− PITAND: - PANDAS (*n* = 3) - unspecified (*n* = 1) − Autoimmune disorder: - PANS (*n* = 2) - AIH (*n* = 1) Autoimmune disorder diagnostics: - retrobulbar neuritis (*n* = 1) - demyelination process suspicion (*n* = 1) − Vision disorder, diplopia (*n* = 1) − Sepsis, tension headache (*n* = 1) − Progressive Duchenne’s muscular dystrophy (*n* = 1) − Cervical spinal cord tumour, femoral shaft fracture (*n* = 1)
anti-PCA2 (*n* = 6)	− PITAND: - EBV associated vocal and motor tics, nocturnal urination (*n* = 1) - unspecified tics, acute neuropsychiatric disorder (*n* = 1) − Epilepsy (*n* = 1) − Autism (*n* = 1) − Peroneal nerves axonopathy, epilepsy (*n* = 1) − Chronic tic disorder, epileptic episodes (*n* = 1)
anti-GAD (*n* = 5)	− Epilepsy (*n* = 2) − Left anterior cerebral artery area ischaemic stroke (*n* = 1) − CIDP (*n* = 1) − Autism, behaviour disorder, developmental retardation (*n* = 1)
anti-Yo (*n* = 6)	− Autism, toxocariasis, paroxysmal anxiety disorder (*n* = 1) − Chronic vocal and motor disorder (*n* = 1) − Demyelination in left frontal lobe lesion, vertigo, bilateral headache, malaise, muscle weakness (*n* = 1) − Post-infectious polyneuropathy, limbs numbness (*n* = 1) − AIE, disorientation, sleep disorder, loss of consciousness (*n* = 1) − Nodular periventricular heterotopy, autoimmune disorder (*n* = 1)
anti-CV2 (*n* = 2)	− Vertigo, balance impairment in streptococcal pharyngitis, antecedent herpetic infection (*n* = 1) − Autism, speech delay, two brothers with PDD (*n* = 1)
anti-Ma/Ta (*n* = 1)	− MS, optic neuritis (*n* = 1)
anti-ganglioside IgG (*n* = 5)	− Neuropathy: - GBS (*n* = 1) - IX, X nerve neuropathy (*n* = 1) - acute axonal polyneuropathy (*n* = 1) − Autism, motor tics, speech retardation, generalised hypotonia (*n* = 1) − Tourette syndrome, IVIG-dependent improvement (*n* = 1)
anti-ganglioside IgM (*n* = 4)	− PANDAS (*n* = 1) − Motor tics, fainting (*n* = 1) − Behaviour disorder, inadequate reaction to stress (*n* = 1) − PITAND susp., involuntary movement disorder
serum anti-NMDAr (*n* = 20)	− Encephalitis suspicion (*n* = 3) − Epilepsy (*n* = 3) − PDD (*n* = 3) − Encephalitis: - autoimmune (*n* = 1) - LE (*n* = 1) − Tics (*n* = 2) − Autoimmune disorder suspicion: - demyelination (*n* = 1) - PDD, behaviour disorder, epilepsy (*n* = 1) − PITAND (*n* = 1) − PITAND suspicion (*n* = 1) − GBS pharyngeal-cervical-brachial variant (*n* = 1) − Brain stem tumour, symptomatic epilepsy, psychomotor delay (*n* = 1) − MRI vascular lesions, speech disorder, stuttering (*n* = 1)
anti-AQP4 (*n* = 1)	− non-specific visual disorders, stress-associated aggravation (*n* = 1)
patients with no anti-ganglioside, anti-AQP4, antineural and anti-NMDAr antibodies (*n* = 343)	− meningitis: - infective (HHV-7 (*n* = 2), influenza A (*n* = 1), HSV-1 + *n*. meningitidis (*n* = 1), non-specified (*n* = 9) encephalitis: - infective (VZV (*n* = 3), HSV-1 (*n* = 2), HHV-6 (*n* = 1), influenza A (*n* = 1), rotavirus (*n* = 1), non-specified (*n* = 2), cerebral toxocariasis (*n* = 1)) - autoimmune (*n* = 12) with meningeal involvement (*n* = 1) - Rasmussen syndrome (*n* = 1) − cerebellitis: - infective (VZV (*n* = 1), EBV (*n* = 1), non-specified (*n* = 5)) - autoimmune (*n* = 2) − encephalomyelitis: - ADEM (*n* = 2) - infective (non-specified, *n* = 1) - autoimmune (*n* = 2) − neuroborreliosis (*n* = 3) − *M. pneumoniae* induced OMS (*n* = 1) − Neuropathy: - GBS (*n* = 10) - CIDP (*n* = 3) - CMT (*n* = 2) - mononeuropathies (*n* = 5) - postinfectious neuropathy (*n* = 4) - other and undescribed (*n* = 3) − PANS/CANS (*n* = 1) − PANDAS (*n* = 21) − CNS demyelination (*n* = 7) − Optic nerve inflammation (*n* = 5) − Vision impairment (*n* = 6) − MS (*n* = 4) − neuromuscular or muscular disease: - myasthenia (*n* = 1) - Duchenne syndrome (*n* = 1) - other diseases and movement disorders (*n* = 10) − PDD: - autism (*n* = 25) - Asperger syndrome (*n* = 2) - other PDD (*n* = 4) − Psychomotor developmental delay (*n* = 10) − Involuntary movement disorder (*n* = 32) − Stroke or TIA (*n* = 3) − Progressive encephalopathy (*n* = 1) − Headache (*n* = 18) − Autoimmune disease suspicion (*n* = 2) − Neoplasms and paraneoplastic syndromes (*n* = 7) − CNS diagnostics (*n* = 20) − Dissociative disorder (*n* = 18) − Epilepsy: - idiopathic generalised (tonic-clonic (*n* = 10), polymorphic (*n* = 6), absence (*n* = 6), myoclonic (*n* = 7), undescribed (*n* = 8)) - idiopathic focal (simple focal (*n* = 6), with generalisation (*n* = 10)) - symptomatic (*n* = 9)

Abbreviations: ADEM—Acute Disseminated Encephalomyelitis; AIE—autoimmune encephalitis; AIH—autoimmune hepatitis; anti-Yo—Purkinje cell cytoplasmic antibody type 1; AQP4—aquaporin-4; ASD—atrial septal defect; CANS—Childhood Acute Neuropsychiatric Symptom; CIDP—Chronic Inflammatory Demyelinating Polyneuropathy; CMT—Charcot-Marie-Tooth Disease; CNS—central nervous system; CSF—cerebrospinal fluid; CT—computed tomography; CV2—collapsin response mediator protein 5; EBV—Epstein–Barr virus; GAD—glutamic acid decarboxylase; GBS—Guillain–Barré syndrome; GFAP—glial fibrillary acidic protein; HHV-6—human herpesvirus type 6; HSV-1—herpes simplex virus type 1; Ig—immunoglobulin; IVIG—intravenous immunoglobulin; JIA—juvenile idiopathic arthritis; LE—limbic encephalitis; M. pneumoniae—Mycoplasma pneumoniae; MAG—myelin-associated glycoprotein; MRI—Magnetic Resonance Imaging; MS—multiple sclerosis NET—neuroendothelium; NMDAr—N-Methyl-D-Aspartate receptor; OCD—obsessive-compulsive disease; OMS—opsoclony-myoclony syndrome; PANDAS—Paediatric Autoimmune Neuropsychiatric Disorders Associated with Streptococcal Infections; PCA2—Purkinje cell cytoplasmic antigen type 2; PDD—pervasive developmental disorder; PITAND—Pediatric Infection-Triggered Autoimmune Neuropsychiatric Disorder; SMA—spinal muscular atrophy; TIA—transient ischemic attack; VZV—varicella zoster virus.

**Table 2:** Median and IQR values of analysed parameters with the division based on detected autoantibodies.

		Age [y/o]	CSF Protein [mg/dL]	CSF Cytosis [/µL]	CSF Glucose [/µL]	CSF Erythrocytes [/µL]	Index	ASO [IU/mL]
	n	median (IQR)	median (IQR)	median (IQR)	median (IQR)	median (IQR)	median (IQR)	median (IQR)
all	508	8.9 (5.4–12.7)	24 (17–38)	2 (1–6)	60 (53–68)	2 (1–24)	0.57 (0.49–0.67)	74 (7–268)
**any positive**	**165**	**8.8 (17–37.5)**	**23 (17–37.5)**	**2 (1–5.5)**	**58 (53–67)**	**2 (1–53)**	**0.57 (0.52–0.67)**	**64 (8–232)**
any antineural	129	8.8 (5.5–12.9)	22 (17–35)	2 (1–4)	57 (52–66)	2 (1–37)	0.56 (0.52–0.66)	64 (7–235)
*anti-Yo*	*6*	*13.3* ^ab^ *(12–16.2)*	*34 (-)*	*2.5 (-)*	*53 (-)*	*3.5 (-)*	*0.57 (-)*	*347 (30–765)*
*anti-CV2*	*3*	*2.5* ^ab^*(2.3–5.7)*	*19.5 (-)*	*1.5 (-)*	*50.5 (-)*	*1500.5 (-)*	*0.55 (-)*	*9 (-)*
*anti-Ma/Ta*	*1*	*12.3 (-)*	*20 (-)*	*13 (-)*		*2 (-)*	*0.75 (-)*	*349 (-)*
*anti-GAD*	*6*	*6.1 (4.4–14.7)*	*26 (6–271)*	*7 (3–10)*	*73 (64–78)*	*1 (1–10)*	*0.73 (-)*	*7 (7–75)*
*anti-MAG*	*15*	*9.2 (7.8–13.7)*	*21 (17–29)*	*2 (1–6)*	*57 (53–67)*	*1 (1–250)*	*0.59 (0.47–0.65)*	*70 (48–235)*
*anti-myelin*	*27*	*7.4 (5.5–13.1)*	*18.5 (17–27)*	*2 (1–3)*	*53.5 ^ab^ (47–59)*	*2 (1–42)*	*0.57 (0.53–0.65)*	*38 (7–309)*
*anti-NET*	*46*	*10.9 (5.3–12.8)*	*24 (17–35)*	*2 (1–4)*	*63 (55–71)*	*3.5 (1–38.5)*	*0.53 (0.52–0.59)*	*63 (7–215)*
*anti-Tr*	*1*	*9.2 (-)*	*18 (-)*	*2 (-)*	*65 (-)*	*1 (-)*	*0.62 (-)*	*711 (-)*
*anti-GFAP*	*27*	*8.9 (5.5–11.8)*	*26 (18–35)*	*2 (1–9)*	*56.5 (48.5–63)*	*1* ^ab^ *(1–1.5)*	*0.58 (0.53–0.66)*	*122.5 (11–638.5)*
*anti-PCA2*	*7*	*4.7* ^ab^ *(2.5–6.7)*	*17 (12–22)*	*1 (1–2)*	*54 (52–59)*	*35 (11–37)*	*0.61 (0.59–0.79)*	*7 (7–57)*
anti-AQP4	1	12.8 (-)	42 (-)	1 (-)	56 (-)	3 (-)	0.55 (-)	167 (-)
IgG anti-ganglioside	8	9.9 (5.9–14.7)	31 (21–76)	3 (1–8)	66 (57–74)	501 (2–2000)	0.79 (0.48–0.84)	51.5 (16.5–119.5)
IgM anti-ganglioside	10	7.5 (5.17–12.6)	21 (15–50)	3.5 (1–7)	49 (46–62.5)	502 (3–1000)	0.56 (0.5–0.72)	64 (26–400)
serum anti-NMDAr	32	9.3 (7.4–13.0)	23 (17–38)	2 (1–4)	62 (53–66)	7.5 (1–81)	0.55 (0.5–0.67)	54 (11–137)
CSF anti-NMDAr	2	11.6 (-)	93.5 ^a^ (-)	22 (-)	56.5 (-)	6003 (-)	0.64 (-)	
**autoantibody-negative**	**343**	**9.0 (5.2–12.6)**	**24 (18–39)**	**2 (1–7)**	**60 (54–69)**	**2 (1–14)**	**0.57 (0.47–0.66)**	**83 (7–281)**

^a^—*p* < 0.05 comparison in the whole population (Mann–Whitney test). ^b^—*p* < 0.05 in the autoantibody-positive population (Mann–Whitney test). Abbreviations: anti-Yo—Purkinje cell cytoplasmic antibody type 1; AQP4—aquaporin-4; ASO—antistreptolysin O; CSF—cerebrospinal fluid; CV2—collapsin response mediator protein 5; GAD—glutamic acid decarboxylase; GFAP—glial fibrillary acidic protein; Ig—immunoglobulin; IQR—interquartile range; IQR—interquartile range; MAG—myelin-associated glycoprotein; NET—neuroendothelium; NMDAr—N-Methyl-D-Aspartate receptor; PCA2—Purkinje cell cytoplasmic antigen type 2.

**Table 3:** The incidence of analysed symptoms with the division based on detected autoantibodies.

	n	Consciousness Loss	Meningeal Signs	Focal Signs	Positive Symptoms	OCD	Headache	Imbalance	Gait Impairment	Paresis	Involuntary Movements	Seizures	Mutism	Impaired Urination	Impaired Defecation	Sight Deterioration
all	508	78 (15.4%)	11 (2.2%)	95 (18.7%)	13 (2.6%)	20 (3.9%)	112 (22%)	49 (9.6%)	59 (11.6%)	86 (16.9%)	118 (23.2%)	89 (17.5%)	10 (2%)	10 (2%)	5 (1%)	78 (15.4%)
**any positive**	**165**	**40 (24.2%)**	**3 (1.8%)**	**31 (18.8%)**	**5 (3%)**	**7 (4.2%)**	**36 (21.8%)**	**14 (8.5%)**	**19 (11.5%)**	**29 (17.6%)**	**44 (26.7%)**	**26 (15.8%)**	**4 (2.4%)**	**2 (1.2%)**	**1 (0.6%)**	**40 (24.2%)**
any antineural	129	33 (25.6%)	3 (2.3%)	26 (20.2%)	2 (1.6%)	3 (2.3%)	29 (22.5%)	11 (8.5%)	15 (11.6%)	21 (16.3%)	31 (24%)	21 (16.3%)	4 (3.1%)	2 (1.6%)	1 (0.8%)	22 (17.1%)
*anti-Yo*	*6*	*2 (33.3%)*	*0 (0%)*	*2 (33.3%)*	*0 (0%)*	*0 (0%)*	*2 (33.3%)*	*0 (0%)*	*2 (33.3%)*	*2 (33.3%)*	*1 (16.7%)*	*0 (0%)*	*0 (0%)*	*0 (0%)*	*0 (0%)*	*0 (0%)*
*anti-CV2*	*3*	*0 (0%)*	*0 (0%)*	*0 (0%)*	*0 (0%)*	*0 (0%)*	*1 (33.3%)*	*0 (0%)*	*1 (33.3%)*	*0 (0%)*	*0 (0%)*	*0 (0%)*	*1 (33.3%)*	*0 (0%)*	*0 (0%)*	*0 (0%)*
*anti-Ma/Ta*	*1*	*0 (0%)*	*0 (0%)*	*0 (0%)*	*0 (0%)*	*0 (0%)*	*1 (100%)*	*0 (0%)*	*0 (0%)*	*0 (0%)*	*0 (0%)*	*0 (0%)*	*0 (0%)*	*0 (0%)*	*0 (0%)*	*1 (100%)*
*anti-GAD*	*6*	*2 (33.3%)*	*0 (0%)*	*3 (50%)*	*0 (0%)*	*0 (0%)*	*2 (33.3%)*	*1 (16.7%)*	*0 (0%)*	*4 (66.7%) ^ab^*	*0 (0%)*	*2 (33.3%)*	*0 (0%)*	*1 (16.7%)*	*1 (16.7%) ^b^*	*1 (16.7%)*
*anti-MAG*	*15*	*0 (0%) ^ab^*	*1 (6.7%)*	*2 (13.3%)*	*0 (0%)*	*0 (0%)*	*2 (13.3%)*	*2 (13.3%)*	*3 (20%)*	*0 (0%)*	*6 (40%)*	*0 (0%)*	*0 (0%)*	*0 (0%)*	*0 (0%)*	*4 (26.7%)*
*anti-myelin*	*27*	*6 (22.2%)*	*1 (3.7%)*	*6 (22.2%)*	*1 (3.7%)*	*0 (0%)*	*5 (18.5%)*	*2 (7.4%)*	*2 (7.4%)*	*6 (22.2%)*	*9 (33.3%)*	*4 (14.8%)*	*2 (7.4%)*	*0 (0%)*	*0 (0%)*	*7 (25.9%)*
*anti-NET*	*46*	*17 (37%) ^ab^*	*1 (2.2%)*	*9 (19.6%)*	*1 (2.2%)*	*3 (6.5%)*	*11 (23.9%)*	*3 (6.5%)*	*5 (10.9%)*	*5 (10.9%)*	*9 (19.6%)*	*11 (23.9%)*	*3 (6.5%) ^a^*	*0 (0%)*	*0 (0%)*	*7 (19.6%)*
*anti-Tr*	*1*	*1 (100%)*	*0 (0%)*	*0 (0%)*	*0 (0%)*	*0 (0%)*	*0 (0%)*	*0 (0%)*	*0 (0%)*	*0 (0%)*	*0 (0%)*	*0 (0%)*	*0 (0%)*	*0 (0%)*	*0 (0%)*	*0 (0%)*
*anti-GFAP*	*27*	*5 (18.5%)*	*0 (0%)*	*5 (18.5%)*	*0 (0%)*	*1 (3.7%)*	*6 (22.2%)*	*4 (14.8%)*	*2 (7.4%)*	*5 (18.5%)*	*5 (18.5%)*	*4 (14.8%)*	*0 (0%)*	*1 (3.7%)*	*0 (0%)*	*2 (7.4%)*
*anti-PCA2*	*7*	*1 (14.3%)*	*0 (0%)*	*0 (0%)*	*0 (0%)*	*0 (0%)*	*0 (0%)*	*0 (0%)*	*1 (14.3%)*	*0 (0%)*	*4 (57.1%)*	*0 (0%)*	*0 (0%)*	*0 (0%)*	*0 (0%)*	*0 (0%)*
anti-AQP4	1	0 (0%)	0 (0%)	0 (0%)	0 (0%)	0 (0%)	1 (100%)	1 (100%)	0 (0%)	0 (0%)	0 (0%)	0 (0%)	0 (0%)	0 (0%)	0 (0%)	1 (100%)
IgG anti-ganglioside	8	2 (25%)	0 (0%)	3 (37.5%)	1 (12.5%)	1 (12.5%)	1 (12.5%)	0 (0%)	3 (37.5%)	5 (62.5%)	3 (37.5%)	0 (0%)	0 (0%)	0 (0%)	0 (0%)	1 (12.5%)
IgM anti-ganglioside	10	4 (40%) ^ab^	0 (0%)	1 (10%)	2 (20%) ^a^	2 (20%) ^a^	1 (10%)	0 (0%)	1 (10%)	2 (20%)	5 (50%) ^a^	1 (10%)	1 (10%)	0 (0%)	0 (0%)	0 (0%)
serum anti-NMDAr	32	4 (12.5%)	0 (0%)	4 (12.5%)	2 (6.3%)	2 (6.3%)	7 (21.9%)	3 (9.4%)	2 (6.3%)	5 (15.6%)	10 (21.3%)	5 (15.6%)	1 (3.1%)	0 (0%)	0 (0%)	8 (25%)
CSF anti-NMDAr	2	2 (100%) ^ab^	0 (0%)	1 (50%)	0 (0%)	0 (0%)	2 (100%) ^ab^	1 (50%)	1 (50%)	0 (0%)	0 (0%)	1 (50%)	0 (0%)	0 (0%)	0 (0%)	1 (50%)
**autoantibody-negative**	**343**	**86 (25.1%)**	**8 (2.3%)**	**64 (18.7%)**	**8 (2.3%)**	**13 (3.8%)**	**76 (22.2%)**	**35 (10.2%)**	**40 (11.7%)**	**57 (16.6%)**	**74 (21.6%)**	**63 (18.4%)**	**6 (1.7%)**	**8 (2.3%)**	**4 (1.2%)**	**50 (14.6%)**

^a^—*p* < 0.05 in the whole population (χ^2^ test), ^b^—*p* < 0.05 in the autoantibody-positive population (χ^2^ test), Abbreviations: anti-gang-anti-ganglioside; anti-Yo—Purkinje cell cytoplasmic antibody type 1; AQP4—aquaporin-4; ASO—antistreptolysin O; CSF—cerebrospinal fluid; CV2—collapsin response mediator protein 5; GAD—glutamic acid decarboxylase; GFAP—glial fibrillary acidic protein; Ig—immunoglobulin; IQR—interquartile range; IQR—interquartile range; MAG—myelin-associated glycoprotein; NET—neuroendothelium; NMDAr—N-Methyl-D-Aspartate receptor; OCD—obsessive-compulsive disorder; PCA2—Purkinje cell cytoplasmic antigen type 2.

**Table 4:** The frequency and positivity percentage in the entire group, in the autoantibody-positive population and with specific antibodies.

		All	Any Ab	*Anti-Yo Ab*	*Anti-CV2 Ab*	*Anti-Ma/Ta Ab*	*Anti-GAD Ab*	*Anti-MAG Ab*	*Anti-myelin Ab*	*Anti-NET Ab*	*Anti-Tr Ab*	*Anti-GFAP Ab*	*Anti-PCA2 Ab*	*Anti-AQP4 Ab*	Anti-gang IgG Ab	Anti-gang IgM Ab	CSF Anti-NMDAr Ab	Serum Anti-NMDAr Ab
sex	female	215 (42.3%)	74 (44.9%)	1 (16.7%)	1 (33.3%)	0 (0%)	4 (66.7%)	3 (20%)	13 (48.1%)	21 (45.7%)	0 (0%)	15 (55.6%)	4 (57.1%)	1 (100%)	2 (25%)	4 (40%)	2 (100%)	16 (50%)
	male	293 (57.7%)	91 (55.1%)	5 (83.3%)	2 (66.7%)	1 (100%)	2 (33.3%)	12 (80%)	14 (51.9%)	25 (54.3%)	1 (100%)	12 (44.4%)	3 (42.9%)	0 (0%)	6 (75%)	6 (60%)	0 (0%)	16 (50%)
living place	rural	208 (40.9%)	60 (36.4%)	0 (0%)	1/3 (33.3%)	0 (0%)	1 (16.7%)	5 (33.3%)	12 (44.4%)	15 (32.6%)	0 (0%)	8 (29.6%)	3 (42.9%)	0 (0%)	2 (25%)	5 (50%)	1 (50%)	16 (50%)
	urban	300 (59.1%)	105 (63.6%)	6 (100%)	2/3 (66.7%)	1 (100%)	5 (83.3%)	10 (66.7%)	15 (55.6%)	31 (67.4%)	1 (100%)	19 (70.4%)	4 (57.1%)	1 (100%)	6 (75%)	5 (50%)	1 (50%)	16 (50%)
anti-HSV1 IgG Ab		103/230 (44.8%)	42/84 (50%)	2/3 (66.7%)	1/2 (50%)	0/1 (0%)	2/3 (66.7%)	3/8 (37.5%)	5/9 (55.6%)	11/24 (45.8%)	-	9/13 (69.2%)	3/6 (50%)	-	3/5 (60%)	2/2 (100%)	2/2 (100%)	7/18 (38.9%)
anti-HSV1 IgM Ab		8/226 (3.5%)	4/82 (4.9%)	0/3 (0%)	1/1 (100%) ^a^	0/1 (0%)	0/3 (0%)	0/8 (0%)	0/8 (0%)	1/25 (4%)	-	0/13 (0%)	0/5 (0%)	-	0/5 (0%)	0/2 (0%)	1/2 (50%)	2/18 (11.1%)
anti-HSV2 IgG Ab		8/229 (3.5%)	6/84 (7.1%)	0/3 (0%)	0/2 (0%)	0/1 (0%)	1/3 (33.3%)	0/8 (0%)	2/9 (22.2%) ^a^	1/24 (4.2%)	-	0/13 (0%)	0/6 (0%)	-	1/5 (20%)	0/2 (0%)	1/2 (50%)	2/18 (11.1%)
anti-HSV2 IgM Ab		1/224 (0.4%)	0/81 (0%)	0/3 (0%)	0/1 (0%)	0/1 (0%)	0/3 (0%)	0/8 (0%)	0/8 (0%)	0/25 (0%)	-	0/13 (0%)	0/5 (0%)	-	0/5 (0%)	0/2 (0%)	0/2 (0%)	0/17 (0%)
anti-EV IgG Ab		67/196 (34.2%)	26/67 (38.8%)	2/3 (66.7%)	0/1 (0%)	1/1 (100%)	1/3 (33.3%)	2/5 (40%)	2/7 (28.6%)	6/21 (28.6%)	-	5/11 (45.5%)	1/3 (33.3%)	-	2/5 (40%)	2/2 (100%)	2/2 (100%)	4/14 (28.6%)
anti-EV IgM Ab		1/194 (0.5%)	1/66 (1.5%)	0/3 (0%)	0/1 (0%)	0/1 (0%)	0/3 (0%)	0/5 (0%)	0/6 (0%)	1/21 (4.8%)	-	0/11 (0%)	0/3 (0%)	-	0/5 (0%)	0/2 (0%)	0/2 (0%)	0/13 (0%)
anti-EBV-VCA IgG Ab		222/367 (60.5%)	82/130 (63.1%)	3/5 (60%)	1/3 (33.3%)	1/1 (100%)	5/6 (83.3%)	9/13 (69.2%)	11/21 (52.4%)	23/38 (60.6%)	-	19/21 (90.5%) ^ab^	4/6 (66.7%)	-	3/7 (42.9%)	4/7 (57.1%)	2/2 (100%)	13/25 (52%)
anti-EBV-VCA IgM Ab		33/368 (9%)	15/130 (11.5%)	1/5 (20%)	1/3 (33.3%)	0/1 (0%)	1/6 (16.7%)	1/13 (7.7%)	3/21 (14.3%)	5/38 (13.2%)	-	3/21 (14.3%)	0/6 (0%)	-	0/7 (0%)	1/7 (14.3%)	1/2 (50%)	5/25 (20%)
EBNA IgG Ab		194/348 (55.7%)	71/124 (57.3%)	2/5 (40%)	1/3 (33.3%)	1/1 (100%)	4/6 (66.7%)	8/13 (61.5%)	10/19 (52.6%)	19/37 (51.4%)	-	17/21 (81%) ^ab^	3/5 (60%)	-	3/6 (50%)	4/7 (57.1%)	1/2 (50%)	11/23 (47.8%)
anti-CMV IgG Ab		159/377 (42.2%)	57/131 (43.5%)	2/6 (33.3%)	0/3 (0%)	1/1 (100%)	3/6 (50%)	9/13 (69.2%) ^a^	9/21 (42.9%)	14/37 (37.8%)	-	11/22 (50%)	3/6 (50%)	-	3/7 (42.9%)	3/6 (50%)	1/2 (50%)	10/24 (41.7%)
anti-CMV IgM Ab		15/377 (4%)	6/131 (4.6%)	0/6 (0%)	0/3 (0%)	0/1 (0%)	1/6 (16.7%)	0/13 (0%)	2/21 (9.5%)	0/37 (0%)	-	0/22 (0%)	0/6 (0%)	-	0/7 (0%)	0/6 (0%)	2/2 (100%) ^ab^	3/24 (12.5%)
anti-TBEV IgG Ab		4/103 (3.9%)	3/33 (9.1%)	0/2 (0%)	-	-	0/2 (0%)	0/1 (0%)	0/1 (0%)	2/15 (13.3%)	-	0/7 (0%)	-	-	1/3 (33.3%)	0/2 (0%)	0/1 (0%)	1/5 (20%)
anti-B. burgdorferi IgG Ab		14/378 (3.7%)	6/124 (4.8%)	0/4 (0%)	0/1 (0%)	0/1 (0%)	0/5 (0%)	1/10 (10%)	0/21 (0%)	3/34 (8.8%)	-	0/19 (0%)	0/5 (0%)	0/1 (0%)	1/8 (12.5%)	0/7 (0%)	0/2 (0%)	1/23 (4.3%)
anti-B. burgdorferi IgM Ab		30/380 (7.9%)	9/124 (7.3%)	0/4 (0%)	0/1 (0%)	0/1 (0%)	1/5 (20%)	0/10 (0%)	2/21 (9.5%)	2/34 (5.9%)	-	0/19 (0%)	0/5 (0%)	0/1 (0%)	1/8 (12.5%)	0/7 (0%)	1/2 (50%)	4/23 (17.4%)
anti-MP IgG Ab		74/366 (20.2%)	29/125 (23.2%)	1/3 (33.3%)	0/1 (0%)	0/1 (0%)	2/5 (40%)	1/10 (10%)	7/23 (30.4%)	6/34 (17.6%)	0/1 (0%)	4/17 (23.5%)	3/6 (50%)	1/1 (100%)	1/7 (14.3%)	1/6 (16.7%)	1/2 (50%)	6/25 (24%)
anti-MP IgM Ab		17/368 (4.6%)	11/126 (8.7%) ^a^	1/3 (33.3%)	0/1 (0%)	0/1 (0%)	0/5 (0%)	1/10 (10%)	4/23 (17.4%) ^a^	1/35 (2.9%)	0/1 (0%)	0/17 (0%)	0/6 (0%)	0/1 (0%)	1/7 (14.3%)	0/6 (0%)	1/2 (50%)	3/26 (11.5%)
anti-MP IgA Ab		10/360 (2.8%)	5/125 (4%)	0/3 (0%)	0/1 (0%)	0/1 (0%)	0/5 (0%)	1/10 (10%)	2/23 (8.7%)	1/34 (2.9%)	0/1 (0%)	0/17 (0%)	0/6 (0%)	0/1 (0%)	1/7 (14.3%)	0/6 (0%)	0/2 (0%)	0/25 (0%)
ANA		114/310 (36.8%)	33/100 (33%)	1/3 (33.3%)	0/2 (0%)	1/1 (100%)	3/5 (60%)	5/10 (50%)	5/17 (29.4%)	8/30 (26.7%)	0/1 (0%)	4/15 (26.7%)	0/3 (0%)	1 (100%)	3/7 (42.9%)	3/6 (50%)	1/2 (50%)	6/20 (30%)
ANCA		45/211 (21.3%)	13/74 (17.6%)	1/3 (33.3%)	0/1 (0%)	-	1/4 (25%)	2/10 (20%)	2/10 (20%)	6/23 (26.1%)	0/1 (0%)	1/11 (9.1%)	0/2 (0%)	0/1 (0%)	1/6 (16.7%)	3/6 (50%) ^a^	1/2 (50%)	2/11 (18.2%)
ASO > 150 IU/ml		124/331 (37.5%)	36/106 (34%)	2/3 (66.7%)	0/1 (0%)	1/1 (100%)	0/3 (0%)	4/11 (36.4%)	8/23 (34.8%)	8/28 (28.6%)	1/1 (100%)	8/16 (50%)	0/5 (0%)	1/1 (100%)	1/4 (25%)	2/7 (28.6%)	-	4/19 (21.1%)
MRI/CT lesions		148/439 (33.7%)	49/148 (33.2%)	2/6 (33.3%)	1/3 (33.3%)	1/1 (100%)	4/6 (66.7%)	6/12 (50%)	7/24 (29.2%)	16/41 (39%)	1/1 (100%)	5/24 (20.8%)	4/6 (66.7%)	0/1 (0%)	1/7 (14.3%)	2/9 (22.2%)	1/2 (50%)	12/31 (38.7%)
EEG		152/407 (37.3%)	48/133 (36.1%)	0/4 (0%)	1/2 (50%)	-	2/5 (40%)	2/8 (25%)	9/24 (37.5%)	13/40 (32.5%)	1/1 (100%)	9/20 (45%)	3/7 (42.9%)	0/1 (0%)	2/5 (40%)	3/9 (33.3%)	2/2 (100%)	14/30 (46.7%)

^a^—*p* < 0.05 in the whole population (χ^2^ test), ^b^—*p* < 0.05 in the autoantibody-positive population (χ^2^ test), Abbreviations: Ab—antibodies; ANA—antinuclear antibody; ANCA—anti-neutrophil cytoplasmic antibody; anti-gang—anti-ganglioside; anti-Yo—Purkinje cell cytoplasmic antibody type 1; AQP4—aquaporin-4; ASO—antistreptolysin O; B. burgdorferi—Borrelia burgdorferi; CMV—cytomegalovirus; CSF—cerebrospinal fluid; CT—computed tomography; CV2—collapsin response mediator protein 5; EBNA—Epstein–Barr nuclear antigen; EBV—Epstein–Barr virus; EBV-VCA—Epstein–Barr virus viral-capsid antigen; EEG—electroencephalography; EV—enterovirus; GAD—glutamic acid decarboxylase; GFAP—glial fibrillary acidic protein; HSV—Herpes simplex virus; Ig—immunoglobulin; Ig—immunoglobulin; IQR—interquartile range; MAG—myelin-associated glycoprotein; MP—Mycoplasma pneumoniae; MRI—Magnetic resonance imaging; NET—neuroendothelium; NMDAr—N-Methyl-D-Aspartate receptor; PCA2—Purkinje cell cytoplasmic antigen type 2; TBEV—tick-borne encephalitis virus.

## Data Availability

Data sharing not applicable.
